# Super-Resolution Imaging of Tight and Adherens Junctions: Challenges and Open Questions

**DOI:** 10.3390/ijms21030744

**Published:** 2020-01-23

**Authors:** Hannes Gonschior, Volker Haucke, Martin Lehmann

**Affiliations:** 1 Leibniz-Forschungsinstitut für Molekulare Pharmakologie (FMP), 13125 Berlin, Germany; gonschior@fmp-berlin.de (H.G.); haucke@fmp-berlin.de (V.H.); 2 Faculty of Biology, Chemistry, Pharmacy, Freie Universität Berlin, 14195 Berlin, Germany

**Keywords:** super-resolution microscopy, structured illumination microscopy, stimulated emission depletion, single molecule localization microscopy, adherens junction, tight junction

## Abstract

The tight junction (TJ) and the adherens junction (AJ) bridge the paracellular cleft of epithelial and endothelial cells. In addition to their role as protective barriers against bacteria and their toxins they maintain ion homeostasis, cell polarity, and mechano-sensing. Their functional loss leads to pathological changes such as tissue inflammation, ion imbalance, and cancer. To better understand the consequences of such malfunctions, the junctional nanoarchitecture is of great importance since it remains so far largely unresolved, mainly because of major difficulties in dynamically imaging these structures at sufficient resolution and with molecular precision. The rapid development of super-resolution imaging techniques ranging from structured illumination microscopy (SIM), stimulated emission depletion (STED) microscopy, and single molecule localization microscopy (SMLM) has now enabled molecular imaging of biological specimens from cells to tissues with nanometer resolution. Here we summarize these techniques and their application to the dissection of the nanoscale molecular architecture of TJs and AJs. We propose that super-resolution imaging together with advances in genome engineering and functional analyses approaches will create a leap in our understanding of the composition, assembly, and function of TJs and AJs at the nanoscale and, thereby, enable a mechanistic understanding of their dysfunction in disease.

## 1. Introduction

### 1.1. Adherens Junctions and Tight Junctions

Epithelial and endothelial cells line multiple organs to segregate internal and external compartments. Cell contacts are formed on the one hand by the adherens junction (AJ) which provides mechanical adhesion, and on the other by the tight junction (TJ) which primarily acts as a barrier to solutes and water and as a fence that separates the apical and basolateral plasma membrane domains. However, some TJ proteins form paracellular channels for small cations, anions, or water [1].

Both junctions are characterized by extracellularly interacting transmembrane proteins that bind via intracellular adapter proteins to the cytoskeleton [2].

TJs were first observed by transmission electron microscopy (TEM) as apical membrane contacts extending over 200–500 nm with electron-dense areas forming a continuous belt-like attachment by fusing the outer membrane leaflets of two neighboring cells into a ≈3 nm thick structure [3]. Later, by using freeze fracture electron microscopy (FFEM) their structure was described as an interconnecting strand network with a general distance between its single strands of 30–80 nm in epithelial cells and tissues [4]. The first protein components of TJs were identified as Zonula occludens (ZO-1) [5] and later the transmembrane proteins occludin [6], junctional adhesion molecule A (JAM-A) [7], and claudins [8]. Occludin, together with tricellulin and marvel-D3 form the tetraspan family of TJ-associated Marvel Domain Proteins (TAMPs) [9]. Claudins regulate interactions between the TAMPs and vice versa [10]. The mammalian genome codes for 27 claudin proteins with specific functions and tissue-specific expression patterns. Zonula occludens 1 and 2 (ZO-1, -2) turned out to be the major adapter proteins on the cytosolic side of TJs [5,11,12]. They act via various interaction domains as scaffolds for occludin, claudins, and other integral TJ proteins, providing their linkage to the actin cytoskeleton [13,14,15,16]. For JAM-A it could be recently shown that its assembly enables epithelial cell polarization. JAM-A is a single transmembrane protein that regulates membrane apposition. In contrast, tetra spanning proteins like claudins form via their two extracellular loops paracellular barriers or channels. So far it could be shown that claudin-2 [17] and claudin-15 [18] promote increased water permeability and both claudins, as well as claudin-10b, also promote increased cation permeability [19,20,21]. Whereas, claudin-10a [21] and claudin-17 [22] contribute to a higher anion permeability. It is also known that some specific claudin combinations can support paracellular ion permeability, e.g., claudin-16/-19 for cations [23] and claudin-4/-8 for small anions [24]. As recently described a knockout of multiple claudins results in increased ion permeability but does not affect macromolecular diffusion or polarity in epithelial cells indicating that other TJ-related proteins must be involved [25].

The initial strand and TJ-meshwork formation is highly dependent on claudins [26] which form via *cis*- and *trans-*interactions arrays of TJ strands which are about 10 nm in diameter. TJ strands assemble to a complex TJ meshwork at bicellular junctions. A specialized site of the TJ where macromolecules can pass has been identified to be the tricellular TJ (tTJ), the location where three cells meet [27]. The proteins of the tTJ are tricellulin [28], and three singe-transmembrane proteins, angulin-1 to -3 [29]. The tTJ is virtually impermeable at high tricellulin expression for macromolecules but becomes permeable for it at low expression as observed in the inflammatory bowel disease ulcerative colitis [30].

The AJ mediates mechanical cell–cell interactions during development and in differentiated tissues. Intercellular adhesion is mediated by *cis*- and *trans*-association of extracellular domains of cadherins coded by 20 different genes in vertebrates. Tissue specific cadherins are epithelial cadherins (E-cad), neuronal cadherin (N-cad), and vascular endothelial cadherins (VE-cad). The *trans*-interactions of E-cad extracellular domains are Ca^2+^ dependent. Intracellularly E-cad are linked via catenin [31,32,33], vinculin [34,35], and other actin-binding proteins to the actin cytoskeleton [36]. Two forms of AJ have been observed, first linear AJ linked to the circumferential actin belt in mature epithelial sheets and second punctuate AJ at free edges with direct actin linkage. Actin bundles in linear AJ can be contracted by actomyosins to produce planar-polarized apical constrictions during tissue remodeling. Rho GTPases, formins, and myosins regulate actin polymerization and contraction. Cadherin can be removed from the surface via clathrin mediated endocytosis (CME) that counterbalances actomyosin mediated constriction in neighboring cells [37]. Therefore, AJs notably integrate both cellular adhesion and cortical tension sensing [38] important for tissue homeostasis and remodeling during development.

In order to mediate cell adhesion and barrier formation, both AJ and TJ form dense and complex multiprotein structures. Some of their characteristics like formation of the TJ meshwork have been visualized by electron microscopy (EM) [4]. However, due to the inherent limitations of EM with respect to defining molecular and structural dynamics many key questions remain unsolved yet: for example, how are the individual protein components like channel and barrier forming claudins of TJs or the different components of AJs organized within the junction and how does their interaction contribute to the overall functionality? How are junctions remodeled, e.g., by strand reformation, endocytosis, during cell division, or upon injury? To answer these questions, it is indispensable to understand the nanoscale organization of the molecular components of TJs (claudins, TAMPs, JAMs, ZOs) and AJs (cadherins, catenins, vinculin, actin-binding proteins) in fixed cells and tissue and, most importantly, in living preparations. Such an approach requires multi-color imaging at nanoscale resolution by super-resolution microscopy (SRM). SRM offers the possibility to perform quantitative analysis of nanoclusters of AJs or measurements of the strand and meshwork organization of TJs.

Recent studies using SRM have begun to uncover the nanoscale organization and dynamics of TJs [13,39,40] and AJs and their components [41,42,43]. Here we provide an overview on recent advances and limitations in SRM, summarize important new insight in junction research from recent studies and provide an outlook for future research.

### 1.2. Resolution Limit

**Figure 1:**
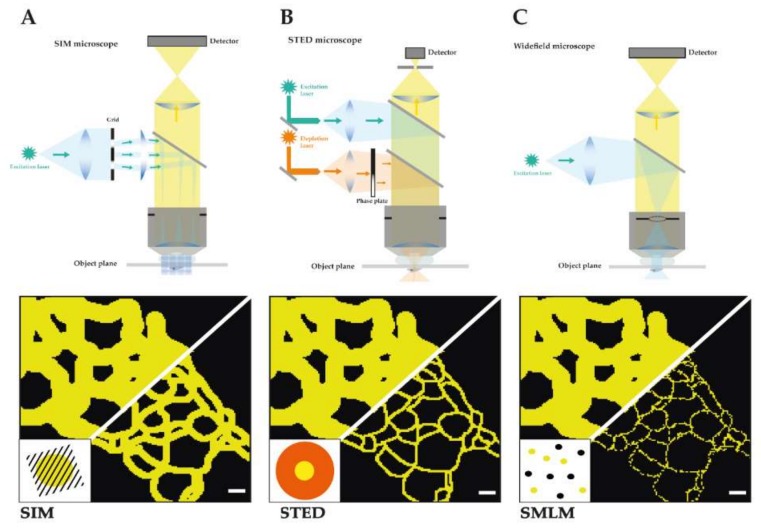
Schematic depiction of the main three SRM techniques. It is shown for each technique a detailed construction of the microscope and the light path as well as a schematic comparison of a reconstituted TJ meshwork resolved by structured illumination microscopy (SIM) (resolution limit: 100 nm) (**A**), time-gated stimulated emission depletion (gSTED) (resolution limit: 40 nm) (**B**), and single molecule localization microscopy (SMLM) (resolution limit: 20 nm) (**C**) in reference to confocal imaged TJ meshwork (resolution limit: 250 nm). Scale bars: 200 nm.

TJs were initially identified by electron microscopy analysis of ultrathin sections of resin embedded cells and tissues as µm-sized apical membrane contacts with characteristic electron dense areas. These membrane contacts appeared to act as a barrier to organic dyes and showed specific membrane kissing points [3]. Using freeze fracture sample preparation these close membrane appositions could be resolved as fence-like intramembrane strands with characteristic mesh sizes of ≈30–80 nm and strand morphology [4]. Using immunogold labelling on resin and freeze fracture samples the early biochemically identified components of TJs, namely claudins and occludin, were shown to be localized to these meshworks and specifically copolymerize in strands [6,8]. Since FFEM involves difficult sample preparation and the immunogold labelling of chemically fixed samples even with overexpressed proteins and with high affinity antibodies is often incomplete, a dense and specific nanoscale distribution of multiple TJ and AJ proteins was not achieved so far. Fluorescence microscopy on the other hand offers high density labelling and highly sensitive detection of AJ and TJ components in intact tissues and living cells. Generally, the resolution of fluorescence microscopes is limited by Abbe’s law to 200–250 nm in the lateral and 500–700 nm in axial direction [44]. Therefore, TJs in polarized epithelia often appear as diffraction-limited spots in x-z scans. Several fluorescence microscopy techniques have overcome the resolution limit by either spatially or temporally controlling fluorescence emission of which structured illumination microscopy (SIM), time-gated stimulated emission depletion (gSTED), and single molecule localization microscopy (SMLM) have turned out to be the three main techniques to visualize biological specimens at the nanoscale (Figure 1).

### 1.3. Structured Illumination Microscopy (SIM)

In structured illumination microscopy (SIM) spatially restricted fluorescence emissions are produced by stripe-patterned wide-field excitation (see Figure 1A) [45]. Multiple camera images are collected from an excitation pattern with different phases and orientations. The collected images contain high resolution information as moiré fringes are formed from the fluorescent sample illuminated by the stripe illumination. The high-resolution information from SIM images can be extracted from Fourier-transformed images in a process called deconvolution. Several illumination modes have been used to create SIM excitation patterns, e.g., in 3D [45], in a total internal reflection mode [46,47] at a grazing incidence angle [48] or in a lattice light sheet [49]. The final SIM images show a lateral resolution of typically 100–130 nm and an axial resolution of 100–250 nm that depends on the wavelength of emission light, the numerical aperture (NA) of the objective, the distance from the coverslip, and the illumination mode. Using an objective with the highest NA or nonlinear SIM with patterned activation resolutions of 84 and 48 nm, respectively, have been achieved [50].

SIM offers key advantages like the use of standard organic fluorophores and fluorescent proteins, low excitation power, and fast imaging speeds up to several hundred frames per second [48]. Since the image reconstruction relies heavily on mathematical processing the point-spread function (PSF) must be determined carefully, and low intensity raw images or high background can produce substantial reconstruction artefacts. Commercial wide-field SIM systems are offered by Zeiss (Elyra S1 and 7), GE Healthcare (Deltavision OMX), and Nikon (N-SIM), while lattice light sheet SIM is offered by 3i.

Point-scanning SIM or Re-Scan Microscopy uses a confocal microscope for scanning an excitation spot over the sample and collects the fluorescence emission directly on a multi-array detector in the absence of a pinhole element in the conjugate plane [51]. The signal at the central pixel and the signal distribution over multiple arrays represent accurately the PSF of the microscope and can be used to increase the resolution by a factor of 1.4 using linear deconvolution. The total signal intensity on the array is then reassigned to the scanned pixel. Since in the point-scanning SIM the pinhole of the confocal microscope is removed more light is collected, the resolution and signal-to-noise is increased by working with an accurately measured PSF and linear deconvolution. Alternatively, in a rescanning mode a camera detects the scanned emission signal with a higher magnification that also results in a 1.4-fold increase in resolution. Commercial systems are available from Zeiss (Airy Detector) and Confocal.nl (RCM) [52].

Standard SIM works best within the first 10 µm from the coverslip that would limit the observation of native TJs and AJs to certain cell types or thin tissues sections. If TJs and AJs form at flat cell contacts, e.g., punctuate AJs that are within 1 µm from the coverslip restricted excitation in total internal refection (TIRF) and GI-SIM would enable to visualize fast dynamics of TJ strands or AJ protein cluster movements with up to 250 fr/s over extended time periods at very low light doses [48]. Additionally, regarding the high detection efficiency and low light input of SIM we would see it as an ideal tool to investigate the AJ/TJ components at near endogenous levels for example by CrispR mediated knock-in of fluorescent tag or in optically cleared samples. Since SIM relies on mathematical image reconstruction with a strong influence of microscope alignment, temperature, and sample parameters, a high fluorescent signal intensity, low background, and careful calibrations are required to produce artefact-free SIM images. Overall SIM enables robust, sensitive, and fast imaging with highest contrast, but only achieves routinely resolution down to 100 nm resolution [53]. This resolution that is approximately half the diffraction limit would not enable resolving of individual TJ strands, small TJ meshes, cadherin nanoclusters, or the cortical actin organization at AJs.

### 1.4. Stimulated Emission Depletion (STED)

In stimulated emission depletion (STED) spatially restricted fluorescence emissions are produced in the center of a coaligned focused excitation spot surrounded by a ring-shaped depletion pattern (see Figure 1B) [54]. The emissions from the diffraction-limited excitation spot is depleted by the ring-shaped depletion focus except for the center region. By scanning the coaligned focused spots and detecting the emission in a confocal microscope, images with a lateral resolution of about 50 nm can be obtained by optimizing imaging conditions such as excitation and depletion energies. The confocal configuration eliminates out of focus light and offers both high contrast and optical sectioning. Pulsed excitation and time-gated detection have enabled resolutions below 50 nm [55]. Using a two-objective configuration or 3D STED depletion patterns a lateral and axially similar resolution of 50–70 nm has been demonstrated [56]. The overall STED resolution is mostly limited by depletion laser power, alignment quality and, most importantly, by labelling density and the availability of bright and photostable dyes with an optimal 5%–10% normalized emission intensity at the depletion laser wavelength. Commercial STED microscopes can be purchased from Leica, Picoquant, or Abberior Instruments.

Since both TJ and AJ are formed by oligomeric transmembrane proteins the lower signal to noise effect of STED can be outweighed by higher label density, e.g., by claudins forming strands by *cis*-and *trans*-interactions. Low abundant or under-labelled structures may not give enough signal for STED imaging. Multicolor STED is routinely performed with 2–3 channels. Therefore, STED imaging of TJs and AJs with optimal labelling [57,58] and optimized 2D and 3D cell culture conditions will show several TJ and AJ components as well as the actin cytoskeleton with <60 nm resolution deep inside cells [59]. Despite STED having the lowest signal to background ratio of all super-resolution techniques it produces raw images that usually do not require post-processing. During a practical comparison STED and SMLM gave similar resolutions on microtubules and vesicular structures [53]. Another advantage of STED is the possibility of imaging cellular structures in living cells and tissues with <50 nm resolution [58] and comparing the ultrastructural preservation after chemical fixation, permeabilization, and immune labelling [60]. Especially, the ultrastructure of tension-sensitive AJ, complex TJ strand morphologies, and the actin cytoskeleton could be altered by chemical fixation. Native AJs and TJs extend in the axial direction in polarized monolayers and are found several µm away from the glass surface. Here the optical sectioning of STED and the possibility to increase resolution in Z direction down to 70 nm would enable the visualization of TJ and AJ structures in a native context. For functional studies of nanoscale dynamics of AJ and TJ protein dynamics STED can be combined with fluorescence recovery after photobleaching (FRAP), fluorescence correlation spectroscopy (FCS), and fluorescence lifetime imaging microscopy (FLIM).

### 1.5. Single Molecule Localization Microscopy (SMLM)

Single Molecule Localization Microscopy (SMLM) increases resolution by exerting temporal control of fluorescence emission. Single molecule fluorescence emissions are detected from a sparse subset of fluorophores that show recovery from an inactive state (see Figure 1C) [61,62], photo activation [63], photo conversion, or short presence in the detection volume [64]. Depending on the brightness of the fluorescence signal the position of the single molecule can be determined with 2–50 nm precision [61,63,65]. The molecule positions are extracted from tens of thousands of images of sparse emitters, drift corrected, and plotted into a new super-resolution image. The SMLM image resolution is determined by both localization precision and localization density [66]. The limited photon output of single molecule emissions often requires strong laser excitation, sensitive detection by low noise cameras, and imaging in TIRF mode within 200 nm from the coverslip. Three-dimensional SMLM was achieved by astigmatic distortion of the PSF in z direction [67], by multi-plane detection [68] or an interferometric approach [69,70]. Both organic fluorophores and photoactivatable or blinking fluorescent proteins can be used as labels for SMLM. The movement of single biological molecules in cells could be analyzed by using tracking SMLM [71]. SMLM offers the highest lateral and axial resolution and can be performed with relatively simple setups when significant challenges of labelling density, sample drift, acquisition time, multicolor acquisition, and image analysis are overcome. A wide range of image analysis packages for 2D and 3D SMLM are available [72]. Commercial SMLM systems are offered by Leica (Leica GSD), Nikon (N-STORM), Zeiss (Zeiss Elyra P1) and Bruker (Vutara 350).

Similar to STED, the SMLM techniques offer diffraction unlimited resolution when high labelling densities can be achieved. Therefore, endogenous labelling of TJ components should be performed with polyclonal antibodies that can bind multiple epitopes. The maximal resolution of SMLM can be in the order of 10 nm that should be sufficient to resolve individual claudin strands or E-cad nanoclusters. Such high resolutions often require direct genetic labelling of proteins of interest with either photoactivatable proteins or self-labelling enzymatic tags. SMLM with photoactivatable fluorescent proteins especially in tissue is often limited by auto fluorescent and detection of rather dim fluorescence bursts and could therefore overestimate monomeric E-cad and underestimate clusters. Therefore, SMLM imaging should be performed close to the cover glass, e.g., in flat endothelial cells or in sparse cultures of epithelial cells that form flat cellular overlaps with TJ strands. Since flat portions of cellular contacts are often not within the typical depth of a TIRF, illumination of ≈200 nm SMLM should be performed with wide-field excitation and 3D SMLM. When 3D SMLM is used on AJ or TJ structures that are located several micrometers away from the glass coverslip the signal to noise ratio decreases that could result in more false-positive localization and a lower localization precision. The higher signal of SMLM in TIRF mode enables the analysis of individual AJ and TJ proteins formed in the lower plasma membrane, e.g., during early steps of polymerization, intracellular anchoring, or signal transduction outside of AJ and TJ. In order to reveal the nanoscale organization of multiple claudins in TJ meshworks or E-cad clusters together with intracellular AJ proteins multicolor SMLM is required. Major challenges for multicolor imaging are first to find spectrally different fluorophores with a similar performance as the widely used Alexa Fluor 647 dye with similar photon outputs, second harmonize buffer requirements of multiple fluorophores, and third register multicolor images after drift correction.

**Figure 2:**
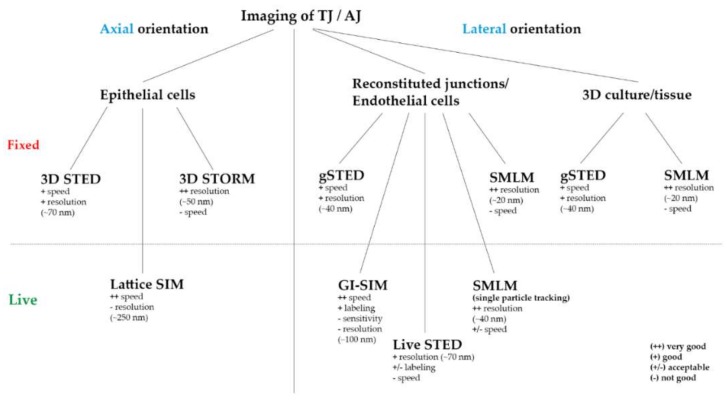
Practicable overview of the ideally used SRM technique for TJ and AJ imaging regarding the axial or lateral direction of the junction, the system (epi-, endothelial cells/fibroblasts/tissue), different labeling (fluorescent proteins/antibodies) and imaging (fixed/live) approaches.

A general technical review [73] and comparison of SIM, STED, and SMLM can be found in [53]. In direct comparison both SMLM and STED gave the highest resolution of <50 nm compared to 110 nm for SIM, but SIM and SMLM offered the highest signal-to-noise ratio [53]. The principle and important considerations for TJ/AJ imaging are summarized in Figure 1, Figure 2 and Table 1.

## 2. Results

### 2.1. Super-Resolution Imaging of Adherens Junctions Reveals Nanoscale Clustering and Stratified Intracellular Organization

AJs form at overlaps of endothelial cells and basolateral of TJs in epithelial cells and integrate tension sensing and adhesion remodeling [74]. In EM images AJs appear as close membrane appositions with an electron dense cytosolic plaque of actin [3] but freeze fracture images show no continuous strands of E-cad molecules. Instead, E-cad molecules were found in individual clusters using diffraction limited fluorescence microscopy [75].

At the nanoscale level endogenous E-cad in *Drosophila* embryos was found to form discrete nanometer-sized clusters by SMLM. The protein density estimations from fluorescence correlation spectroscopy calibrations and from single molecule counting were consistent with tightly packed E-cad molecules about eight nanometers apart from each other. Most E-cad was monomeric with varying cluster sizes that have been found to be regulated by dynamin-mediated endocytosis. Conversely, elimination of α-catenin or PAR3 reduced E-cad clustering, while interfering with action polymerization [41].

Similarly, when SMLM 3D-STORM was performed in epithelial cells with both bright organic dyes and photoactivatable proteins, highly homogenous cluster sizes of 50–60 nm with a fixed distance of ≈160 nm were identified [42]. E-cad clusters in epithelial cells contained six molecules similar to results in *Drosophila* embryos [41] and form independently of cadherin–cadherin interactions. Mutation in E-cad that interfered with *trans* or *cis*-interaction still produced clusters, albeit with a decreased molecular density. Coculture experiment found coexisting adhesive and non-adhesive E-cad clusters with similar size. Dual color SMLM revealed that adhesive and non-adhesive E-cad clusters are delimited by F-actin. Both the actin depolymerizing drug Latrunculin A and expression of a tail-less E-cad results in the formation of larger E-cad clusters. Therefore, SMLM revealed that E-cad forms a nanocluster surrounded by an actin fence that depends both on homophilic interactions and anchoring via the cytosolic tail. Even larger and more mature AJs are formed by groups of individual nanoclusters that can be linked to and stabilized by intracellular scaffold proteins [42]. Therefore, adhesion and tension sensing are mediated by small units of E-cad that are evenly spaced and can be finely regulated by incorporation into larger units and controlled by dynamin-dependent endocytosis.

How are the intracellular proteins of AJs organized? Using a combination of 3D PALM, surface-generated structured illumination together with biochemical perturbation on planarized biomimetic cadherin-based AJs the nanoscale architecture of AJ was analyzed [43]. Notably a plasma membrane-proximal cadherin-catenin compartment segregated from the actin cytoskeletal compartment, connected by an interface zone containing vinculin was found. The position of vinculin is determined by catenin and vinculin showed a tension and tyrosine phosphorylation dependent extended confirmation. The robust surface-generated structured illumination assay revealed the nanoscale changes of vinculin conformation in different cell types, under biochemical and pharmacological perturbations and could be verified by fluorescence resonance energy transfer (FRET)-tension measurements. Therefore, vinculin integrates mechanical and chemical signals between cadherin and the actomyosin layer to control cell adhesion in cells and tissues [43].

Of note, a similar stratified nanoarchitecture and tension sensitive conformation of vinculin was previously found in focal adhesions (FA) [76]. Integrins form nanoclusters [77] while the tension sensing molecules paxillin and vinculin form linear and non-colocalizing areas within FA [78]. Vinculin FRET-based tension sensors revealed a high tension especially at small FA and that a general increase in cellular tension increases the size of FA [79].

### 2.2. Super-Resolution Imaging Shows Claudin Meshworks, Strand Dynamics, and Molecular Composition of the TJ

TJs are organized by claudin strands and meshworks that were revealed by FFEM of cell and tissue samples. Since EM requires strong fixation and often lacks molecular specificity SRM would be needed to confirm the nanoarchitecture of TJ in intact cells and tissues.

A first approach in this direction was done in 2003 when Sasaki et al. were able to image dynamics of claudin strands and whole TJ meshworks in claudin-1-GFP overexpressing L-fibroblasts using confocal microscopy. However, due to the previously described resolution limit a further analysis of multiple claudins and strand dynamics in denser not peripheral meshwork areas was not possible [80].

**Figure 3:**
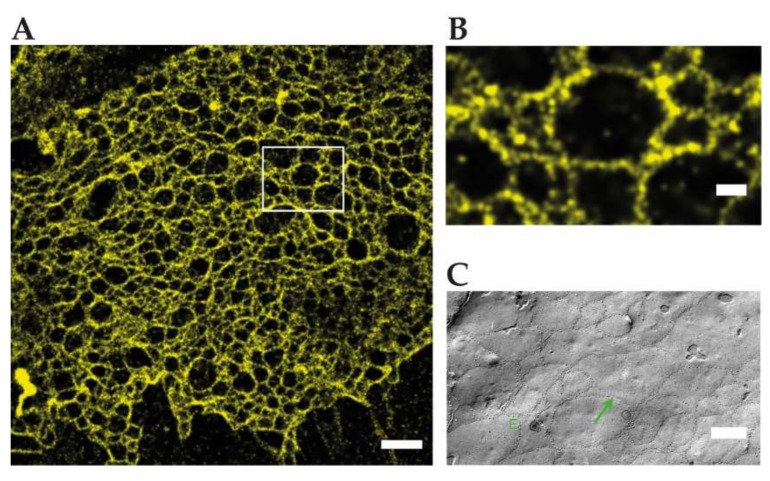
Reconstituted TJ networks formed by claudin3-YFP in HEK293 cells imaged by using SMLM (adapted from Kaufmann et al. 2012 [39]); (**A**) localization microscopy image of the region marked in the conventional wide-field fluorescence image with a mean effective optical resolution 48 nm. Scale bar: 1 µm; (**B**) magnification of claudin-3-YFP in HEK293 cells. Scale bar: 200 nm; (**C**) FFEM of claudin-5-YFP/claudin-3-YFP cotransfected HEK293 cells. Scale bar: 250 nm.

Van Itallie et al. reconstituted eGFP-claudin-2 strands in fibroblasts and imaged extended strands networks by live SIM with ≈120 nm resolution. Large meshes as well as single or double strand configurations and strand dynamics could be detected. Claudin-2 networks formed in the absence of claudin-2-ZO-1 binding and showed a higher mobility and failed to co-align with the actin cytoskeleton. Using multicolor SIM, a partial overlap between actin, ZO-1 and claudin-2 could be found, since actin and ZO-1 are also highly concentrated at AJ. Strand breaks and strand elongation occurred independently of ZO-1 binding, but preferentially at branch points. Branch points were also identified as polymerization sites of claudin and showed a weak enrichment of occludin. How other claudins copolymerize, associate with actin, ZO-1, and occludin are important questions for the nanostructure and the regulation of TJs. Since some claudins like claudin-3 form much smaller meshes in reconstituted TJ meshworks (e.g., Figure 3C) than claudin-2, super-resolution techniques with higher spatial resolution like SMLM and STED will help to resolve these questions [13].

Kaufmann et al. reconstituted TJs from C-terminally YFP tagged claudin-3 and claudin-5 in an epithelial cell line, devoid of endogenous claudin expression. SMLM with 50 nm structural resolution resolved meshworks and found two meshwork populations with ≈100/360 nm (claudin-3) and ≈60/480 nm (claudin-5) mesh sizes. Notably inhomogeneous claudin distribution along strands and in claudin proteins between strands were detected and could play a role in strand formation. Since SMLM requires >20,000 frames for image reconstruction, the dynamics of claudin meshworks were not resolved. Available multicolor SMLM could resolve mixtures of claudins or the nanoscale organization of TJ proteins in the future [39] (Figure 3).

An example for TJs resolved with STED microscopy has not been published yet. Just Cording et al. in 2017 were able to image changes of the Tricellular nanoarchitecture upon treatment with tricellulin derived peptides [81].

**Figure 4:**
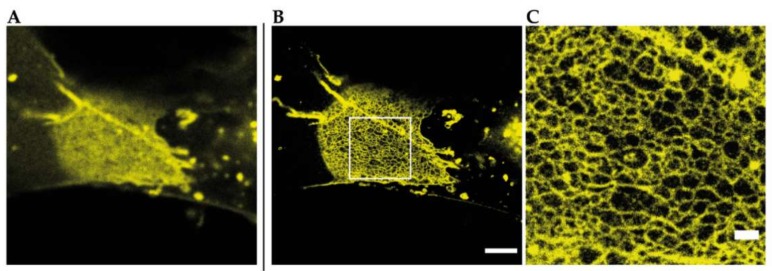
Reconstituted TJ networks formed by YFP-claudin-3 in Cos7 fibroblasts (unpublished data) imaged by using STED microscopy; (**A**) confocal overview image of a reconstituted TJ meshwork at the overlapping area of two transfected cells; (**B**) STED image of the same meshwork area from A. Scale bar: 2 µm; (**C**) magnification of the marked area in B. Scale bar: 500 nm.

That STED microscopy in general is a tool to resolve TJ meshworks at nanoscale level without any further image processing is shown in Figure 4 by an example of a reconstituted meshwork of YFP-claudin-3 in Cos7 fibroblasts.

On an endogenous level, the group around Schlingmann and Koval studied the effect of alcohol on the nanostructure of alveolar epithelial tight junction using SMLM in combination with functional readouts. Alcohol increased claudin-5 levels, induced claudin-18 and actin containing spikes, and increased the paracellular flux. SMLM revealed mostly linear nanocluster arrangements of immunolabelled claudin-5, -18, and ZO1 in TJs. Under alcohol treatment, the specific nano-colocalization of claudin-18 and ZO1 decreased, while the colocalization of claudin-5 and claudin-18 increased. As molecular mechanism leading to alcohol induced paracellular flux the competition of claudin-5 and claudin-18 for interaction with ZO1 was proposed [40].

## 3. Discussion and Outlook

Multicellular organisms and tissues rely on multiple cellular junction structures to mediate adherence, sense tension, and form a paracellular barrier. AJs and TJs form dense and complex multiprotein assemblies with a specific nanoscale architecture. While EM revealed claudin strands and membrane kissing points at the TJ or dense cytosolic plaques at the AJ, the intricate molecular nano-organization and dynamic remodeling of these cellular structures only begin to emerge. As shown by SMLM, E-cad forms in the AJ regularly spaced nanoclusters connected to a stratified intracellular layer of tension-sensing and actin interacting proteins at the AJ [41,42,43]. Revealing the dynamic nanoscale organization of AJs, especially movements of cadherins cluster, vinculin conformational changes, and actin remodeling under tension, during vascular development and endothelial tissue remodeling, should give important new insights into AJ regulation. Here, a combination of single particle tracking, and live STED could enable the study of the concerted action of multiple AJ components.

With recently developed, and now well matured, super-resolution microscopy techniques a first glimpse at claudin strand organization and dynamics at TJ was revealed. Since most TJs contain multiple claudins and other TJ associated proteins, e.g., occludin, or JAM, and various TJ-related scaffolding proteins that exchange on second timescales, fast multicolor imaging with nanoscale resolution would be required. How multiple claudins and occludin assemble into the TJ meshwork is still an open question. Since multicolor SMLM microscopy is still challenging because of slow image acquisition, low photon counts from fluorescent proteins, fluorophore incompatibilities, channel registration errors, and drift correction errors in the range of the resolution alternative multicolor techniques like STED and SIM are advantageous. STED imaging could also be applied to living cells and tissues [58]. Special care must be taken for the optimization of fixation, permeabilization, and labelling protocols as cellular nanostructures can be significantly altered during these sample preparation steps [60]. Ideally similar nanostructures should be visible in live STED of genetically tagged proteins, in fixed cells, and after endogenous labelling by antibodies.

Since real TJs in cultured epithelial cells and tissues mostly extend in the axial direction, the significantly lower z-resolution of most available super-resolution techniques sets a barrier to resolving the native TJ nano-architecture. Therefore, TJ components, namely claudins and occludin, were visualized in reconstituted flat cell culture systems that recapitulate early stages of epithelial formation. Here, claudin meshwork structures were visualized that were very similar to early FFEM studies. Importantly any observation in such a reconstituted system would have to be tested in endothelial or epithelial cells, in 3D culture models [59] and, importantly, in tissues. Nevertheless the improvement of SRM techniques which provide imaging at high resolution in thicker samples (>30 µm) and most importantly also with a sufficient z-resolution to image single strands at 50–100 nm spacing in tissue and epithelial cells will be one of the main and most important steps towards understanding the physiology and biology of TJs. One way to achieve this goal will be by using carefully aligned and refractive index matched 3D STED imaging of endogenously tagged abundant TJ proteins, like claudins, ZO-1, or occludin, providing novel insights about claudin localization within the meshwork but also its organization in the lateral membrane. Endogenous labelling still depends on the availability of specific antibodies or recently available genetic knock-in technology. Since SRM techniques resolve fluorophore positions down to a few nanometers, the sizes of antibodies become a limiting factor. Therefore, we expect that super-resolution imaging of endogenous tagged TJ, AJ, and actin will reveal the important nano-structural features of native junctions together with the associated cytoskeleton. Small, bright, and photostable fluorescent labels should be placed as close as possible to biological target structures that can be achieved via genetic tags like fluorescent proteins or self-labelling enzymes like SNAP or Halo tags [82]. Brighter fluorogenic dyes [83], more sensitive detectors, and microscopy systems like light sheet microscopes would enable faster and better resolved imaging of AJ and TJ structures. A better understanding of nano-structural details of TJs and AJs will ultimately enable discovery of new strategies to overcome or strengthen the intercellular barrier for drug delivery or during tissue damage, respectively.

## Figures and Tables

**Table 1:** Overview and direct comparison of the key parameters of SIM, gSTED, and SMLM.

	SIM	gSTED	SMLM
**Resolution limit**	100 nm (lateral) 250 nm (axial)	40 nm (lateral) 70 nm (axial)	20 nm (lateral) 50 nm (axial)
**Multi-color**	4 colors	2–4 colors	2–4 colors
**Sample preparation**	Standard	Standard	Standard
**Live imaging**	Yes	Yes	Yes
**Imaging speed**	10 ms–10 s	10 s–5 min	10 min
**Illumination power**	10–100 W/cm^2^	1–100 kW/cm^2^	1–100 kW/cm^2^

## Abbreviations

AJ	Adherens junction
dSTORM	Direct stochastic optical reconstruction microscopy
E-cad	E-cad
eGFP	Enhanced green fluorescent protein
EM	Electron microscopy
FA	Focal adhesion
FCS	Fluorescence correlation spectroscopy
FFEM	Freeze fracture electron microscopy
fr	Frame
FRAP	Fluorescence recovery after photobleaching
FRET	Fluorescence resonance energy transfer
gSTED	Gated stimulated emission depletion
JAM-A	Junctional adhesion molecule A
kW	Kilowatt
min	Minute
ms	Millisecond
NA	Numerical aperture
nm	Nanometer
PALM	Photoactivated localization microscopy
PSF	Point spread function
s	Second
SIM	Structured illumination microscopy
SMLM	Single molecule localization microscopy
SRM	Super-resolution microscopy
TAMP	Tight junction-associated MARVEL protein
TEM	Transmission electron microscopy
TJ (bTJ, tTJ)	Tight junction (bicellular TJ, tricellular TJ)
YFP	Yellow fluorescent protein
ZO-1, -2, -3	Zonula occludens protein 1, 2, or 3

## References

[1] Günzel D., Fromm M. (2012). Claudins and other tight junction proteins. Compr. Physiol..

[2] Zihni C., Mills C., Matter K., Balda M.S. (2016). Tight junctions: From simple barriers to multifunctional molecular gates. Nat. Rev. Mol. Cell Biol..

[3] Farquahr M.G., Palade G.E. (1963). Junctional complexes in various epithelia. J. Cell Biol..

[4] Staehelin L.A. (1973). Further observations on the fine structure of freeze-cleaved tight junctions. J. Cell Sci..

[5] Stevenson B.R., Siliciano J.D., Mooseker M.S., Goodenough D.A. (1986). Identification of ZO-1: A high molecular weight polypeptide associated with the tight junction (Zonula Occludens) in a variety of epithelia. J. Cell Biol..

[6] Furuse M., Hirase T., Itoh M., Nagafuchi A., Yonemura S., Tsukita S., Tsukita S. (1993). Occludin: A novel integral membrane protein localizing at tight junctions. J. Cell Biol..

[7] Martìn-Padura I., Lostaglio S., Schneemann M., Williams L., Romano M., Fruscella P., Panzeri C., Stoppacciaro A., Ruco L., Villa A. (1998). Junctional adhesion molecule, a novel member of the immunoglobulin superfamily that distributes at intercellular junctions and modulates monocyte transmigration. J. Cell Biol..

[8] Furuse M., Fujita K., Hiiragi T., Fujimoto K., Tsukita S. (1998). Claudin-1 and -2: Novel integral membrane proteins localizing at tight junctions with no sequence similarity to occludin. J. Cell Biol..

[9] Raleigh D.R., Marchiando A.M., Zhang Y., Shen L., Sasaki H., Wang Y., Long M., Turner J.R. (2010). Tight Junction–associated MARVEL Proteins MarvelD3, Tricellulin, and Occludin Have Distinct but Overlapping Functions. Mol. Biol. Cell.

[10] Cording J., Berg J., Kading N., Bellmann C., Tscheik C., Westphal J.K., Milatz S., Gunzel D., Wolburg H., Piontek J. (2013). In tight junctions, claudins regulate the interactions between occludin, tricellulin and marvelD3, which, inversely, modulate claudin oligomerization. J. Cell Sci..

[11] Jesaitis L.A., Goodenough D.A. (1994). Molecular characterization and tissue distribution of ZO-2, a tight junction protein homologous to ZO-1 and the Drosophila discs-large tumor suppressor protein. J. Cell Biol..

[12] Balda M.S., Gonzalez-Mariscal L., Matter K., Cereijido M., Anderson J.M. (1993). Assembly of the tight junction: The role of diacylglycerol. J. Cell Biol..

[13] Van Itallie C.M., Tietgens A.J., Anderson J.M. (2017). Visualizing the dynamic coupling of claudin strands to the actin cytoskeleton through ZO-1. Mol. Biol. Cell.

[14] Hirokawa N., Tilney L.G. (1982). Interactions between actin filaments and between actin filaments and membranes in quick-frozen and deeply etched hair cells of the chick ear. J. Cell Biol..

[15] Madara J.L., Pappenheimer J.R. (1987). Structural basis for physiological regulation of paracellular pathways in intestinal epithelia. J. Membr. Biol..

[16] Fanning A.S., Jameson B.J., Jesaitis L.A., Anderson J.M. (1998). The tight junction protein ZO-1 establishes a link between the transmembrane protein occludin and the actin cytoskeleton. J. Biol. Chem..

[17] Rosenthal R., Milatz S., Krug S.M., Oelrich B., Schulzke J.D., Amasheh S., Günzel D., Fromm M. (2010). Claudin-2, a component of the tight junction, forms a paracellular water channel. J. Cell Sci..

[18] Rosenthal R., Günzel D., Piontek J., Krug S.M., Ayala-Torres C., Hempel C., Theune D., Fromm M. (2019). Claudin-15 forms a water channel through the tight junction with distinct function compared to claudin-2. Acta Physiol..

[19] Amasheh S., Meiri N., Gitter A.H., Schöneberg T., Mankertz J., Schulzke J.D., Fromm M. (2002). Claudin-2 expression induces cation-selective channels in tight junctions of epithelial cells. J. Cell Sci..

[20] Van Itallie C.M., Fanning A.S., Anderson J.M. (2003). Reversal of charge selectivity in cation or anion-selective epithelial lines by expression of different claudins. Am. J. Physiol. Ren. Physiol..

[21] Van Itallie C.M., Rogan S., Yu A., Vidal L.S., Holmes J., Anderson J.M. (2006). Two splice variants of claudin-10 in the kidney create paracellular pores with different ion selectivities. Am. J. Physiol. Ren. Physiol..

[22] Fromm M., Günzel D., Krug S.M., Amasheh S., Rosenthal R., Schulzke J.D., Fromm A., Conrad M.P. (2012). Claudin-17 forms tight junction channels with distinct anion selectivity. Cell. Mol. Life Sci..

[23] Hou J., Renigunta A., Konrad M., Gomes A.S., Schneeberger E.E., Paul D.L., Waldegger S., Goodenough D.A. (2008). Claudin-16 and claudin-19 interact and form a cation-selective tight junction complex. J. Clin. Investig..

[24] Hou J., Renigunta A., Yang J., Waldegger S. (2010). Claudin-4 forms paracellular chloride channel in the kidney and requires claudin-8 for tight junction localization. Proc. Natl. Acad. Sci. USA.

[25] Otani T., Nguyen T.P., Tokuda S., Sugihara K., Sugawara T., Furuse K., Miura T., Ebnet K., Furuse M. (2019). Claudins and JAM-A coordinately regulate tight junction formation and epithelial polarity. J. Cell Biol..

[26] Furuse M., Sasaki H., Fujimoto K., Tsukita S. (1998). A single gene product, claudin-1 or -2, reconstitutes tight junction strands and recruits occludin in fibroblasts. J. Cell Biol..

[27] Krug S.M., Amasheh S., Richter J.F., Milatz S., Günzel D., Westphal J.K., Huber O., Schulzke J.D., Michael F. (2009). Tricellulin Forms a Barrier to Macromolecules in Tricellular Tight Junctions without Affecting Ion Permeability. Mol. Biol. Cell.

[28] Ikenouchi J., Furuse M., Furuse K., Sasaki H., Tsukita S., Tsukita S. (2005). Tricellulin constitutes a novel barrier at tricellular contacts of epithelial cells. J. Cell Biol..

[29] Higashi T., Tokuda S., Kitajiri S.I., Masuda S., Nakamura H., Oda Y., Furuse M. (2013). Analysis of the ‘angulin’ proteins LSR, ILDR1 and ILDR2—Tricellulin recruitment, epithelial barrier function and implication in deafness pathogenesis. J. Cell Sci..

[30] Krug S.M., Bojarski C., Fromm A., Lee I.M., Dames P., Richter J.F., Turner J.R., Fromm M., Schulzke J.D. (2018). Tricellulin is regulated via interleukin-13-receptor α2, affects macromolecule uptake, and is decreased in ulcerative colitis. Mucosal Immunol..

[31] Huber A.H., Weis W.I., West C.D. (2001). The Structure of the ß-Catenin/E-Cadherin Complex and the Molecular Basis of Diverse Ligand Recognition by ß-Catenin. Cell.

[32] Pokutta S., Weis W.I. (2000). Structure of the Dimerization and ß-Catenin-Binding Region of a-Catenin. Mol. Cell.

[33] Drees F., Pokutta S., Yamada S., Nelson W.J., Weis W.I. (2005). α-Catenin Is a Molecular Switch that Binds E-Cadherin-β-Catenin and Regulates Actin-Filament Assembly. Cell.

[34] Watabe-Uchida M., Uchida N., Imamura Y., Nagafuchi A., Fujimoto K., Uemura T., Vermeulen S., Van Roy F., Adamson E.D., Takeichi M. (1998). α-Catenin-Vinculin Interaction Functions to Organize the Apical Junctional Complex in Epithelial Cells. J. Cell Biol..

[35] Weiss E.E., Kroemker M., Rüdiger A., Jockusch B.M., Rüdiger M. (1998). Vinculin Is Part of the Cadherin–Catenin Junctional Complex: Complex Formation between α-Catenin and Vinculin. J. Cell Biol..

[36] Kobielak A., Fuchs E., Medical H.H. (2004). α-Catenin: At the junction of intercellular adhesion and actin dynamics. Nat. Rev. Mol. Cell Biol..

[37] Nanes B.A., Chiasson-MacKenzie C., Lowery A.M., Ishiyama N., Faundez V., Ikura M., Vincent P.A., Kowalczyk A.P. (2012). P120-Catenin Binding Masks an Endocytic Signal Conserved in Classical Cadherins. J. Cell Biol..

[38] Lecuit T., Yap A.S. (2015). E-cadherin junctions as active mechanical integrators in tissue dynamics. Nat. Cell Biol..

[39] Kaufmann R., Piontek J., Grüll F., Kirchgessner M., Rossa J., Blasig I.E., Cremer C. (2012). Visualization and Quantitative Analysis of Reconstituted Tight Junctions Using Localization Microscopy. PLoS ONE.

[40] Schlingmann B., Overgaard C.E., Molina S.A., Lynn K.S., Mitchell L.A., Dorsainvil White S., Mattheyses A.L., Guidot D.M., Capaldo C.T., Koval M. (2016). Regulation of claudin/zonula occludens-1 complexes by hetero-claudin interactions. Nat. Commun..

[41] Quang B.A.T., Mani M., Markova O., Lecuit T., Lenne P.F. (2013). Principles of E-cadherin supramolecular organization in vivo. Curr. Biol..

[42] Wu Y., Kanchanawong P., Zaidel-Bar R. (2015). Actin-Delimited Adhesion-Independent Clustering of E-Cadherin Forms the Nanoscale Building Blocks of Adherens Junctions. Dev. Cell.

[43] Bertocchi C., Wang Y., Ravasio A., Hara Y., Wu Y., Sailov T., Baird M.A., Davidson M.W., Zaidel-bar R., Toyama Y. (2017). Nanoscale architecture of cadherin-based cell adhesions. Nat. Cell Biol..

[44] Abbe E. (1873). Beiträge zur Theorie des Mikroskops und der mikroskopischen Wahrnehmung. Arch. Mikrosk. Anat..

[45] Schermelleh L., Carlton P.M., Haase S., Shao L., Winoto L., Kner P., Burke B., Cardoso M.C., Agard D.A., Gustafsson M.G.L. (2008). Subdiffraction multicolor imaging of the nuclear periphery with 3D structured illumination microscopy. Science.

[46] Chung E., Kim D., So P.T.C. (2006). Extended resolution wide-field optical imaging: Internal reflection fluorescence microscopy. Opt. Lett..

[47] Guo M., Chandris P., Giannini J.P., Trexler A.J., Fischer R., Chen J., Vishwasrao H.D., Rey-Suarez I., Wu Y., Wu X. (2018). Single-shot super-resolution total internal reflection fluorescence microscopy. Nat. Methods.

[48] Guo Y., Li D., Zhang S., Yang Y., Liu J.J., Wang X., Liu C., Milkie D.E., Moore R.P., Tulu U.S. (2018). Visualizing Intracellular Organelle and Cytoskeletal Interactions at Nanoscale Resolution on Millisecond Timescales. Cell.

[49] Chen B.C., Legant W.R., Wang K., Shao L., Milkie D.E., Davidson M.W., Janetopoulos C., Wu X.S., Hammer J.A., Liu Z. (2014). Lattice light-sheet microscopy: Imaging molecules to embryos at high spatiotemporal resolution. Science.

[50] Li D., Shao L., Chen B.C., Zhang X., Zhang M., Moses B., Milkie D.E., Beach J.R., Hammer J.A., Pasham M. (2015). Extended-resolution structured illumination imaging of endocytic and cytoskeletal dynamics. Science.

[51] De Luca G.M.R., Breedijk R.M.P., Brandt R.A.J., Zeelenberg C.H.C., de Jong B.E., Timmermans W., Azar L.N., Hoebe R.A., Stallinga S., Manders E.M.M. (2013). Re-scan confocal microscopy: Scanning twice for better resolution. Biomed. Opt. Express.

[52] Huff J. (2015). The Airyscan detector from ZEISS: Confocal imaging with improved signal-to-noise ratio and super-resolution. Nat. Methods.

[53] Wegel E., Göhler A., Lagerholm B.C., Wainman A., Uphoff S., Kaufmann R., Dobbie I.M. (2015). Imaging cellular structures in super-resolution with SIM, STED and Localisation Microscopy: A practical comparison. Sci. Rep..

[54] Hell S.W., Wichmann J. (1994). Breaking the diffraction resolution limit by stimulated emission: Stimulated-emission-depletion fluorescence microscopy. Opt. Lett..

[55] Vicidomini G., Moneron G., Han K.Y., Westphal V., Ta H., Reuss M., Engelhardt J., Eggeling C., Hell S.W. (2011). Sharper low-power STED nanoscopy by time gating. Nat. Methods.

[56] Curdt F., Herr S.J., Lutz T., Schmidt R., Engelhardt J., Sahl S.J., Hell S.W. (2015). isoSTED nanoscopy with intrinsic beam alignment. Opt. Express.

[57] Grimm J.B., Muthusamy A.K., Liang Y., Brown T.A., Lemon W.C., Patel R., Lu R., Macklin J.J., Keller P.J., Ji N. (2017). A general method to fine-tune fluorophores for live-cell and in vivo imaging. Nat. Methods.

[58] Bottanelli F., Kromann E.B., Allgeyer E.S., Erdmann R.S., Baguley S.W., Sirinakis G., Schepartz A., Baddeley D., Toomre D.K., Rothman J.E. (2015). Two-colour live-cell nanoscale imaging of intracellular targets. Nat. Commun..

[59] Maraspini R., Wang C.-H., Honigmann A. (2020). Optimization of 2D and 3D cell culture to study membrane organization with STED microscopy. J. Phys. Appl. Phys..

[60] Schnell U., Dijk F., Sjollema K.A., Giepmans B.N.G. (2012). Immunolabeling artifacts and the need for live-cell imaging. Nat. Methods.

[61] Rust M.J., Bates M., Zhuang X. (2006). Sub-diffraction-limit imaging by stochastic optical reconstruction microscopy (STORM). Nat. Methods.

[62] Bates M., Huang B., Dempsey G.T., Zhuang X. (2007). Multicolor super-resolution imaging with photo-switchable fluorescent probes. Science.

[63] Betzig E., Patterson G.H., Sougrat R., Lindwasser O.W., Olenych S., Bonifacino J.S., Davidson M.W., Lippincott-Schwartz J., Hess H.F. (2006). Imaging intracellular fluorescent proteins at nanometer resolution. Science.

[64] Giannone G., Hosy E., Levet F., Constals A., Schulze K., Sobolevsky A.I., Rosconi M.P., Gouaux E., Tampe R., Choquet D. (2010). Dynamic superresolution imaging of endogenous proteins on living cells at ultra-high density. Biophys. J..

[65] Vaughan J.C., Jia S., Zhuang X. (2012). Ultrabright photoactivatable fluorophores created by reductive caging. Nat. Methods.

[66] Jones S.A., Shim S.H., He J., Zhuang X. (2011). Fast, three-dimensional super-resolution imaging of live cells. Nat. Methods.

[67] Huang B., Wang W., Bates M., Zhuang X. (2008). Three-Dimensional Super-Resolution Reconstruction Microscopy. Science.

[68] Juette M.F., Gould T.J., Lessard M.D., Mlodzianoski M.J., Nagpure B.S., Bennett B.T., Hess S.T., Bewersdorf J. (2008). Three-dimensional sub-100 nm resolution fluorescence microscopy of thick samples. Nat. Methods.

[69] Shtengel G., Galbraith J.A., Galbraith C.G., Lippincott-Schwartz J., Gillette J.M., Manley S., Sougrat R., Waterman C.M., Kanchanawong P., Davidson M.W. (2009). Interferometric fluorescent super-resolution microscopy resolves 3D cellular ultrastructure. Proc. Natl. Acad. Sci. USA.

[70] Xu K., Babcock H.P., Zhuang X. (2012). Dual-objective STORM reveals three-dimensional filament organization in the actin cytoskeleton. Nat. Methods.

[71] Manley S., Gillette J.M., Patterson G.H., Shroff H., Hess H.F., Betzig E., Lippincott-Schwartz J. (2008). High-density mapping of single-molecule trajectories with photoactivated localization microscopy. Nat. Methods.

[72] Sage D., Pham T.A., Babcock H., Lukes T., Pengo T., Chao J., Velmurugan R., Herbert A., Agrawal A., Colabrese S. (2019). Super-resolution fight club: Assessment of 2D and 3D single-molecule localization microscopy software. Nat. Methods.

[73] Schermelleh L., Ferrand A., Huser T., Eggeling C., Sauer M., Biehlmaier O., Drummen G.P.C. (2019). Super-resolution microscopy demystified. Nat. Cell Biol..

[74] Maître J.L., Berthoumieux H., Krens S.F.G., Salbreux G., Jülicher F., Paluch E., Heisenberg C.P. (2012). Adhesion functions in cell sorting by mechanically coupling the cortices of adhering cells. Science.

[75] Adams C.L., Nelson W.J., Smith S.J. (1996). Quantitative analysis of cadherin-catenin-actin reorganization during development of cell-cell adhesion. J. Cell Biol..

[76] Case L.B., Baird M.A., Shtengel G., Campbell S.L., Hess H.F., Davidson M.W., Waterman C.M. (2015). Molecular mechanism of vinculin activation and nanoscale spatial organization in focal adhesions. Nat. Cell Biol..

[77] Van Zanten T.S., Cambi A., Koopman M., Joosten B., Figdor C.G., Garcia-Parajo M.F. (2009). Hotspots of GPI-anchored proteins and integrin nanoclusters function as nucleation sites for cell adhesion. Proc. Natl. Acad. Sci. USA.

[78] Shroff H., Galbraith C.G., Galbraith J.A., White H., Gillette J., Olenych S., Davidson M.W., Betzig E. (2007). Dual-color superresolution imaging of genetically expressed probes within individual adhesion complexes. Proc. Natl. Acad. Sci. USA.

[79] Grashoff C., Hoffman B.D., Brenner M.D., Zhou R., Parsons M., Yang M.T., McLean M.A., Sligar S.G., Chen C.S., Ha T. (2010). Measuring mechanical tension across vinculin reveals regulation of focal adhesion dynamics. Nature.

[80] Sasaki H., Matsui C., Furuse K., Mimori-Kiyosue Y., Furuse M., Tsukita S. (2003). Dynamic behavior of paired claudin strands within apposing plasma membranes. Proc. Natl. Acad. Sci. USA.

[81] Cording J., Arslan B., Staat C., Dithmer S., Krug S.M., Krüger A., Berndt P., Günther R., Winkler L., Blasig I.E. (2017). Trictide, a tricellulin-derived peptide to overcome cellular barriers. Ann. N. Y. Acad. Sci..

[82] Liss V., Barlag B., Nietschke M., Hensel M. (2015). Self-labelling enzymes as universal tags for fluorescence microscopy, super-resolution microscopy and electron microscopy. Sci. Rep..

[83] Lukinavičius G., Umezawa K., Olivier N., Honigmann A., Yang G., Plass T., Mueller V., Reymond L., Corrêa I.R., Luo Z.G. (2013). A near-infrared fluorophore for live-cell super-resolution microscopy of cellular proteins. Nat. Chem..

